# When Left Is One and Right Is Double: An Experimental Investigation of Visual Allesthesia after Right Parietal Damage

**DOI:** 10.3390/vision4010016

**Published:** 2020-03-01

**Authors:** Denise Baumeler, Sabine Born, Nicolas Burra, Radek Ptak

**Affiliations:** 1 Cognitive Psychology, Faculty of Psychology and Educational Sciences, University of Geneva, 1205 Geneva, Switzerland; denise.baumeler@unige.ch (D.B.); sabine.a.hild@gmail.com (S.B.); nicolas.burra@unige.ch (N.B.); 2 Division of Neurorehabilitation; Department of Clinical Neurosciences; Geneva University Hospital, 1206 Geneva, Switzerland; 3 Laboratory of Cognitive Neurorehabilitation, Faculty of Medicine, University of Geneva, 1206 Geneva, Switzerland

**Keywords:** visual illusion, allesthesia, palinopsia, polyopsia, visual cortex, parietal lobe

## Abstract

Illusory visual phenomena, such as palinopsia, polyopsia or allesthesia, are rare manifestations of posterior cortical damage. Symptoms are characterized by illusory perceptions, ranging from isolated stationary objects to scenes and moving persons. Such illusions may appear while the original object is still in view, or become manifest with a delay and last for minutes, hours or even days. Some authors have suggested a disinhibited cortical response underlying visual illusions, but experimental studies supporting this hypothesis are lacking. Here, we examined a rare patient who after focal right parietal injury consistently reported a second stimulus on the left when briefly shown a target in his right hemifield. The patient perceived the illusory stimulus as less intense, and therefore concluded that it must have a different shape than the original stimulus. A masking experiment revealed that the frequency of the illusion was inversely related to the visibility of the original stimulus, suggesting that it depended on early, feedforward visual processing. We propose that illusory perceptions reflect the interplay of two physiological processes: a fast and automatic activation of contralateral, homotopic visual cortex after unilateral stimulation, and the lack of top-down inhibition following damage to the posterior parietal cortex.

## 1. Introduction

Illusions or hallucinations are rare visual phenomena in patients with focal brain damage. They make up a family of visual symptoms—including palinopsia, polyopsia and visual allesthesia—which have in common the persistence or perseveration of the original stimulus [1,2,3]. Palinopsia is the persistence for seconds, minutes or even days of visual impressions of an entire stimulus or parts of it [4,5]. In patients with visual field impairment the illusory stimulus may be perceived in the intact hemifield (in which case it overlaps with, or adds to another stimulus), or within the scotoma [6,7]. Palinoptic images can be simple (dots, lines) or quite complex, such as a letter, a car registration number, a beard, or a dog walking by [8,9,10]. Some authors have retained abnormal aftereffects as plausible explanation of palinoptic phenomena [11]. However, the latter are distinct from after-images in terms of duration, intensity and quality. After-images are generally quickly erased by new incoming stimulation, are faint and have colors that are complementary to the original image. In contrast, palinoptic images have an appearance and duration that do not depend on the intensity of the original image, are often in the same colors as the original (or in black and white), can be multiple, and may follow a moving object, or in the direction of or against an eye movement [3,6,12].

Polyopsia is characterized by repeated images of a stimulus or its parts, which are seen after the patient fixates the original stimulus for some time and appear while it is still in view [13,14]. Polyopsia may be mistaken for diplopia, as the illusory image is generally close to the original one, and patients may sometimes describe their vision as blurred. While polyopsia may be associated with visual field impairment and movements of gaze in a specific direction [15], in some patients the phenomenon is monocular and therefore of peripheral origin [16,17].

The third illusory phenomenon—visual allesthesia—is less well described than the previous two. The primary characteristic of allesthesia is that patients perceive a sensation as occurring at a position remote from where the stimulus actually is, or extending beyond the original stimulus [1]. There is, however, considerable overlap of allesthesia with palinoptic or polyoptic sensations. For example, patients may see the original stimulus and an illusory stimulus in the opposite hemifield that persists long after disappearance of the original [18], or they may report that the illusory stimulus alternatively appears and disappears [19].

There are currently no satisfactory explanations of these visual illusions. Though many authors likened them to visual afterimages, they also emphasized the differences between illusions and afterimages [4,6,12]. The main reason for the lack of knowledge of the cognitive and physiological mechanisms underlying illusory perceptions is the scarcity of experimental studies of these phenomena.

We here present an experimental assessment of a patient with isolated right posterior parietal damage following a hemorrhagic stroke, who consistently reported the illusory perception of a contralesional stimulus when shown an ipsilateral item. We evaluated the consistency and perceptual quality of this illusion as well as effects of spatial attention and the impact of metacontrast masking on its presence. Our findings suggest that this illusion is triggered by a fast, feedforward response of the visual cortex to a new onset, and that it occurs due to absence of inhibitory feedback from the parietal lobe.

## 2. Case Report

**Figure 1:**
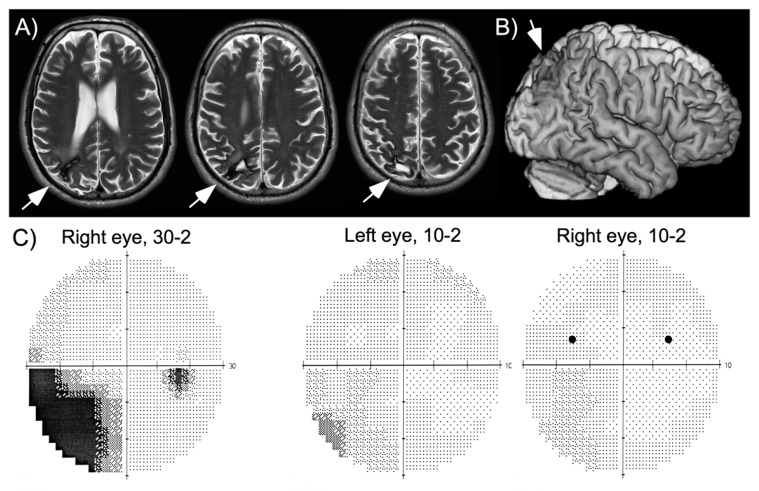
Magnetic resonance imaging (MRI) scan and visual field plot of the patient. (**A**) Axial slices at three different levels of the parietal lobe (images shown according to radiological convention, left is on the right). (**B**) Three-dimensional reconstruction of the patient’s brain, showing the small lesion in the superior parietal cortex. (**C**) Visual field plots (Humphrey Perimeter; 30-2 plot: sensitivity tested up to 30 degrees into the left and right hemifield; 10-2 plots: sensitivity tested for the central 10 degrees; inter-stimulus distance was always two degrees). Testing of the sensitivity up to 30 degrees in the periphery shows incomplete left lower quadrantanopia. More detailed testing of the central 10 degrees shows intact central visual fields. The two black dots indicate positions where targets were presented.

HG is a 60-year-old, well-educated man who suffered a right parietal hemorrhage four years before this study. He had a first neurological and neuropsychological examination within a week after his stroke, which showed partial sensory loss of the left arm, left inferior quadrantanopia, moderate spatial neglect and visual-constructive deficits. While hospitalized, the patient had a series of partial epileptic seizures whose focus was localized to the right parietal lobe. He quickly recovered arm sensation and part of his deficits of spatial cognition, though he was unable to take up driving and work due to persistent fatigue. At the time of this study his neuropsychological assessment revealed a slight, but significant bias on line bisection, visual-constructive deficits as well as increased reaction times to stimuli shown in his left visual hemifield and in visual search (Table 1), consistent with his report of slight spatial neglect in activities of daily living (e.g., orienting in new environments, bumping occasionally into people on his left, searching for his keys). A magnetic resonance imaging (MRI) scan performed two years after his hemorrhage showed local damage in the superior part of the posterior parietal cortex and underlying white matter (Figure 1). The patient was seen three years following his hemorrhage for a routine examination. At this time, he still had partial left inferior quadrantanopia, though the central ~10° of vision were preserved (Figure 1). He had been without seizures for more than two years, but continued to receive anticonvulsant medication (carbamazepine).

Illusory perception was detected when we tested extinction on a PC screen with a simple paradigm (black disks shown unilaterally or bilaterally for 176 ms). Our expectation was that HG would be capable of detecting unilateral stimuli in both hemifields, but would eventually fail to report the left stimulus when a competitive item appeared simultaneously on the right (which would correspond to an extinction phenomenon). Preliminary testing showed that the patient always saw a stimulus presented in the right hemifield, but also reported an additional stimulus at the mirror position in his left hemifield. This illusory contralesional stimulus appeared and disappeared simultaneously with the target, could be seen with the left or right eye and had similar size as the target.

The patient gave written informed consent before participating in this study, and our investigation was approved by the Ethical Commission of the Canton of Geneva.

## 3. General Methods

**Apparatus.** All paradigms were created in MATLAB (The MathWorks Inc., Natick, MA, USA) using the Psychophysics Toolbox extensions (Brainard, 1997; Cornelissen, Peters, & Palmer, 2002). Stimuli were displayed on a 21-inch CRT monitor (NEC MultiSync FE2111SB; resolution: 1280 × 1024 pixels; refreshment: 85 Hz), on grey background (RGB-values: 151, 151, 151; 30 cd/m^2^). A chin- and headrest was used to assure a stable viewing distance of 55 cm. Experiments were conducted in a dimly lit room.

### 3.1. Experiment 1. Temporal and Spatial Characteristics of Illusory Perception

Experiment 1 established the presence of illusory perception and examined whether it was present in the upper and lower visual field. In addition, we tested whether illusory images depended on the duration of presentation of stimuli in the right hemifield.

#### 3.1.1. Methods and Procedure

The target was a black disk of 0.5° in diameter (0 cd/m^2^), presented at 5 degrees eccentricity from the center of the screen. Left targets were thus always shown in the intact part of the visual field. The superior visual fields were examined with targets at 3.5 degrees in the upper quadrants, and two separate experiments were run that differed regarding presentation duration (35 ms or 176 ms). The inferior quadrants were tested only with the 35 ms duration, and targets were shown at 3.5 degrees below the horizontal line of view. To ensure lateralized presentation and exclude compensatory eye movements, we monitored gaze throughout the experiments with a remote eye-tracker (Eyelink 1000, SR Research Ottawa, Ottawa, ON, Canada). The eye-tracker was coupled to the computer that delivered the stimuli so that trials in which the patient’s gaze was not within the central 2 × 2 degrees were excluded from analysis.

Throughout each block a central fixation point (0.1° in diameter) was continuously present. Each trial started with a variable fixation interval, followed by one of four target display types: unilateral left, unilateral right, bilateral or absent. The patient was asked to provide one of four corresponding answers about the target location verbally and without time limitation. The interval between the offset of the response display and the onset of the next fixation display was 800 ms. There were 50 trials for each unilateral condition, 80 trials for the bilateral condition and 20 catch trials. They were presented randomly, separated in two blocks of 100 trials each.

#### 3.1.2. Results and Discussion

For stimuli presented in the upper quadrants errors (failure to maintain fixation) accounted for 26 trials (13%) at the 35 ms presentation and for seven trials (3.5%) at the 176 ms presentation. The patient made 100% correct responses at both durations (176 ms and 35 ms) when stimuli were presented in the left hemifield, bilaterally, or were absent. In contrast, when the target was shown in the right upper quadrant at 176 ms, he made 39 (81.2%) correct responses, but reported bilateral targets in eight trials (16.7%) and a unilateral left target in one trial (2.1%). When stimuli were shown at 35 ms his answer was ‘right’ in only eight trials (17.8%), while in the remaining 37 trials (82.2%) he reported a bilateral target. HG reported significantly more illusory stimuli (‘bilateral’ responses in unilateral right trials) at the short compared to the long presentation (χ^2^ = 39.97, *p* < 0.0001).

For targets shown in the inferior quadrants 27 trials (13.5%) were excluded due to fixation loss. In catch trials the patient always denied seeing anything (100% correct). On unilateral left presentations he reported a left target in only 13 trials (29.6%), while he saw bilateral targets in three trials (6.8%), and, in the remaining 28 trials (63.6%), did not see anything. In bilateral trials he made 62 (93.9%) correct responses and indicated seeing only a right stimulus in the remaining four trials (6.1%). Finally, in the critical unilateral right condition he reported a right target in only one trial (2.2%), while in the remaining 45 trials (97.8%) he had the impression of seeing two targets. The proportion of illusory stimuli was significantly higher for the lower compared to the upper visual field (Fisher exact test, *p* < 0.05).

**Figure 2:**
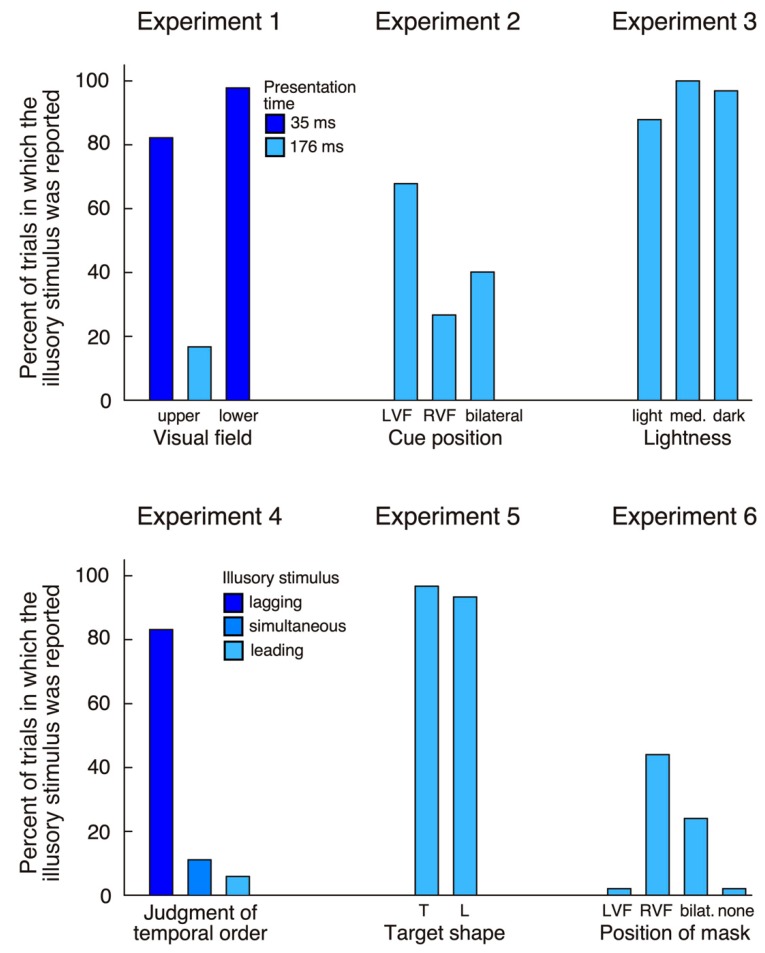
Summary of the findings of all six experiments. Vertical bars show the percentage of responses when HG reported the presence of a stimulus in the left visual field, as a function of the type of stimulus shown in his right visual field. Experiment 1: right target shown in the upper (35 ms and 176 ms) or lower visual field (35 ms); experiment 2: right target, with cue in the left/right visual field or bilateral cues; experiment 3: right target at three levels of lightness; experiment 4: right target; experiment 5: right target is a T or L; experiment 6: right target with left mask, right mask, bilateral mask or no mask (LVF/RVF: left/right visual field).

The main finding of experiment 1 is that, for stimuli shown in his intact right hemifield, HG reported seeing a second stimulus in his left hemifield. This observation was true whether targets were presented in the upper or the lower quadrants (Figure 2). The patient never reported a left stimulus in target absent conditions, indicating that his illusory perception crucially depended on the presence of a target in the right hemifield. Further, although he appeared to have intact visual sensitivity at positions in the upper and lower left quadrant where targets were presented (as shown in his visual field plots, Figure 1), his performance for stimuli in the left inferior quadrant was lower than in the superior quadrant. This is contrary to many cases of palinopsia and polyopsia, who report illusory stimuli within visual scotomas. Another difference is the lack of persistence of the illusory image: while palinopsias and polyopsias typically appear and persist (often for seconds or even minutes) after disappearance of the stimulus that triggers illusory perception, here the illusory phenomenon was short-lasting, simultaneous with the right-sided target and more frequent at very short presentations. All these observations suggest a different underlying mechanism. One possibility is that HG used a conscious strategy, and that he inferred the presence of a left stimulus whenever his perception of the right stimulus was weak (in particular when stimuli were presented at 35 ms). This explanation becomes plausible if we assume that the patient is less confident about his sensations of stimuli shown in the left hemifield when stimuli are shown close to threshold. In the next experiment, we examined whether HG’s illusory perception was modulated by his focus of attention.

### 3.2. Experiment 2. Effects of Spatial Cueing

A brief, peripheral cue improves detection and identification of a probe stimulus presented at the cued (valid) position compared to the uncued (invalid) position [27,28]. There is a wide consensus that this effect reflects a fast shift of attention to the cued location, thus accelerating the processing of an upcoming target. Experiment 2 examined whether illusory perception of our patient was influenced by his focus of attention. The rationale for this experiment was that, if the illusory contralesional stimulus was due to a low-level (sensory) mechanism, the focus of covert attention should not affect its presence. Alternatively, if the illusory stimulus was perceived less frequently when attention was directed to the left hemifield than when it was directed away, this would suggest a higher-level process.

#### 3.2.1. Methods and Procedure

All stimuli were presented in the upper quadrants. The apparatus and procedure were the same as in experiment 1, except that the target (35 ms) was preceded by a 47 ms cue display with an interstimulus interval of 106 ms. The cue consisted of two filled circles of 0.1° in diameter presented above and below the target position (i.e., 2.5° and 4.5°) at 5° left or right of fixation. Cues and targets were either unilateral left, unilateral right or bilateral. All combinations of cue position and target position were used, so that the presence of the target could not be deduced from the presence of a cue. The patient completed 405 trials in a single session, separated into four blocks (45 trials per condition).

#### 3.2.2. Results and Discussion

Unilateral left targets were detected correctly in 100% of all trials whether the cue was in the left or the right hemifield. Similarly, when targets were bilateral the patient made a high number of correct (bilateral) responses (127 trials or 94.1%). These findings indicate that the patient had no difficulty distinguishing the target from the cue. We next examined whether the cue side (left, right, bilateral) would influence his illusory perception of an additional stimulus in the left hemifield when a single right target was presented (Figure 2). When the cue was in the left hemifield, he reported a bilateral target in 31 trials (68.9%), while, when cue and target were in the right hemifield, he only saw an illusory stimulus in 12 trials (26.7%). When cues were bilateral, the frequency of illusory stimuli lay in between (18 trials, 40%). The difference between left cue and right cue conditions was highly significant (χ^2^ = 16.1, *p* < 0.0001).

Thus, cue presentation significantly affected HG’s illusory perception. One observation is that drawing his attention to the left increased the frequency of illusory stimuli. However, even if his attention was drawn to the right hemifield briefly prior to target appearance, in a quarter of all trials he still had the subjective impression of seeing a second target in the contralateral hemifield.

### 3.3. Experiment 3. Perceptual Quality of the Illusory Stimulus

We next tested whether the illusory stimulus had the same perceptual quality for HG as the (real) right stimulus. We presented stimuli to the patient at three different levels of lightness and asked him to evaluate the intensity of his perceptions.

#### 3.3.1. Methods and Procedure

The method and procedure were the same as in experiment 1 except that stimuli were presented at three different levels of gray: dark (4.69 cd/m^2^), intermediate (11.84 cd/m^2^) and light (18.73 cd/m^2^). There were thus three different types of targets on the left or right, as well as all combinations between the three lightness levels for bilateral stimuli. It is important to note that the contrast between target and background was maximal in the dark condition, and, therefore, that the saliency of dark targets was the highest. The patient was asked to report the presence of a target, and should two targets be seen, to indicate whether their lightness was the same or whether one of the two stimuli appeared darker compared to the other. There were 495 trials separated into three blocks, resulting in 33 trials per condition.

#### 3.3.2. Results and Discussion

The patient detected most targets in unilateral left trials (94 correct, 94.9%) and bilateral trials (292 correct, 98.3%), while in almost all unilateral right trials he claimed seeing two items (94 of 99 trials, 94.9%; Figure 2).

In order to evaluate HG’s ability to compare stimulus lightness across hemifields, we first examined his judgment of bilateral conditions. When the left stimulus was darker than the right stimulus, he made a correct judgment in 75 trials (75.8%). Conversely, when the right stimulus was darker than the left stimulus, he correctly judged luminosity in 90 trials (90.9%). When both stimuli were of comparable lightness, HG saw them as equiluminant in only 14 trials (14.1%), while in 59 trials (59.6%) he continued to judge the left stimulus as lighter than the right.

We next evaluated HG’s perception of the illusory stimulus by comparing the unilateral right condition where the stimulus had intermediate lightness to the bilateral conditions where the lightness level of the right stimulus was also intermediate (because only these stimuli could be paired with a stimulus with higher, lower or equal lightness). In the bilateral condition HG perceived the right stimulus as being darker (i.e., more intense) in 71 trials (71.7%). In contrast, he almost always judged the unilateral right stimulus as darker than the illusory left stimulus in 32 trials (97%; Fisher’s exact test, *p* < 0.01). Thus, HG perceived the illusory stimulus in his left hemifield consistently as being of lower intensity than the right stimulus, and this tendency was statistically stronger than what would be expected based on his comparison of bilateral stimuli.

### 3.4. Experiment 4. Temporal Order Judgment

The first three experiments showed that HG consistently perceived an additional stimulus in the contralesional hemifield when a stimulus was shown in his right visual field, though this illusory stimulus was subjectively lighter than the right item. Since the quality of the illusion was subjectively different than the real stimulus, we wondered whether he perceived both as appearing simultaneously or in sequential order.

#### 3.4.1. Methods and Procedure

The methods and procedure were the same as in experiment 1, except that bilateral stimuli were presented concurrently (0 ms delay) or with the left or right leading by 60 ms, 90 ms or 120 ms. The patient was asked to indicate: first, whether he had seen one left, one right or two stimuli and second, should two stimuli be seen, to specify whether they were shown synchronously or whether the left or right stimulus was leading. HG completed 306 trials (34 trials per condition).

#### 3.4.2. Results and Discussion

The patient identified all stimuli in unilateral left (34 trials, 100%) and bilateral trials (238 trials, 100%), while in unilateral right trials he indicated seeing two stimuli in 31 trials (91.2%). His temporal order judgment was correct in 99 trials (97.1%) when the left stimulus was leading or when the right stimulus was leading. He judged simultaneous stimuli correctly as synchronous in 16 trials (47.1%), but in 17 trials (50%) thought that the right stimulus was leading. In the critical unilateral right condition, he judged that the illusory left stimulus was lagging behind the right stimulus in 28 trials (82.4%), was leading in two trials (5.9%) and was simultaneous in four trials (11.8%; Figure 2).

Thus, when shown two stimuli in slight asynchrony, HG had an excellent capacity to judge the temporal order (left first vs. right first) across visual hemifields. When the two stimuli were shown simultaneously he exhibited a slight bias toward judging the right stimulus as appearing first. He showed an even greater bias in his temporal order judgment when shown unilateral right targets; in most of these trials, he judged that the illusory left stimulus lagged behind the right stimulus. Experiment 4 and the preceding experiment 3 thus revealed some information about the subjective phenomenology of the illusory stimulus: it appears less intense and is slightly delayed relative to the right stimulus. Patients with palinopsia or polyopia typically describe the illusory stimulus as being visually similar to the stimulus that triggers the illusory perception. In the next experiment, we therefore explored whether HG could provide information about the shape of the illusory stimulus. 

### 3.5. Experiment 5. Shape of the Illusory Stimulus

In experiment 5, we presented simple letter shapes (L or T) instead of disks and asked HG to indicate the presence of stimuli in the left and right hemifield as well as their identity. Based on observations of palinopsia and polyopsia patients, we expected the left illusory stimulus to have the same shape as the stimulus shown in his right visual field.

#### 3.5.1. Methods and Procedure

The methods and procedure were the same as in experiment 1, except that target stimuli were letters L or T formed by the same horizontal/vertical lines (length, 0.5°). There were four unilateral conditions (L or T, presented to the left or right) and four bilateral conditions (L—L; T—T; L—T; T—L). Stimuli were presented for 35 ms and the patient was asked to indicate their position and identity. There were 240 trials separated into two blocks (30 trials per condition). 

#### 3.5.2. Results and Discussion

HG identified 51.7% targets shown in his left visual field correctly, which is not different from chance performance (χ^2^ > 1.1, *p* > 0.3). In contrast, his performance was well above chance for targets in his right hemifield (95%; Fisher’s exact test, *p* < 0.0001). These findings reflect residual weakness of HG’s left hemifield, which may have been less evident in the detection tasks performed in the previous experiments. The important finding of experiment 5 was that HG again indicated seeing an additional stimulus in the left hemifield in both unilateral right conditions (57 trials, or 95%; Figure 2). In only six of these trials (10.5%), he indicated that the illusory stimulus had the same shape as the letter in the right hemifield, while in 13 trials (22.8%) he was unable to identify it, and in the remaining 38 trials (66.7%) he declared having seen the alternative letter on the left. HG’s tendency to indicate the alternative letter rather than the same letter as presented in his right hemifield was highly significant (χ^2^ = 37.9, *p* < 0.05).

Thus, contrary to the frequent observation that patients with palinopsia and polyopsia perceive the visual perseveration of a stimulus shown in their intact visual field, HG always reported the alternative stimulus. This finding suggests that his identification of the illusory stimulus was based on inferential processes, rather than a low-level sensory mechanism. In order to further test this hypothesis, we examined in the last experiment whether the perception of the illusory stimulus depends on awareness of the stimulus in the right hemifield. 

### 3.6. Experiment 6. Masking Effects

Visual metacontrast masking is a phenomenon characterized by reduction of the visibility of an object caused by the appearance of a second object. This is based on the idea that masking competes with the feedback (‘reentrant’) representation of the probe from higher visual areas, but leaves the early feedforward components unharmed [29]. Given that reentrant processing is responsible for conscious perception of a stimulus, masking decreases the probe’s visibility [30]. Metacontrast masking is strong evidence that visual perception does not depend on pure feedforward flow of information, but that recurrent processing is necessary for conscious perception of the stimulus [31]. However, the mask does not eliminate the early, feedforward representation of the stimulus, which may therefore continue to affect visual processing in tasks that do not depend on conscious awareness of the stimulus. Experiment 6 was intended to determine whether HG’s illusory perception depended on awareness of the stimulus in his right visual field (i.e., later, recurrent processing), or whether it was triggered by early, feedforward signals. 

#### 3.6.1. Methods and Procedure

The target (red disk; 9.69 cd/m^2^, 0.5° in diameter) was presented in the left and/or right upper quadrant (5 degrees left/right of fixation; 3.5 degrees above the horizontal line). In 75% of all trials, the target was masked by a metacontrast mask (blue ring; 9.64 cd/m^2^, of 0.7° in diameter) while in the remaining 25% of trials, it was unmasked. The target was shown for 35 ms, and the mask appeared simultaneously and remained on the screen for 300 ms. Targets were shown in the left hemifield, the right hemifield, or both. The presence and position of the mask varied orthogonally with target position, so that each target condition was equally often paired with a mask shown in the left or right hemifield, both hemifields, or no mask. The patient was asked to report the presence of the target (left, right, bilateral or absent). There were 600 trials separated into five blocks, with 50 trials per condition.

#### 3.6.2. Results and Discussion

We first examined whether metacontrast masking was successful, that is, whether the patient was less likely to report masked compared to unmasked stimuli. For left items, HG showed a robust effect of masking (sum of masked left and masked bilateral vs. sum of masked right and unmasked, 62% vs. 99%, Fisher’s exact test: *p* < 0.0001). The effect of masking for right stimuli was even stronger (sum of masked right and masked bilateral vs. sum of masked left and unmasked, 11% vs. 97%, *p* < 0.0001). Finally, masking was also effective for bilateral stimuli (masked bilateral vs. unmasked, 4% vs. 32%, *p* < 0.0001), but the performance here was much lower even when stimuli were unmasked.

We next examined whether HG’s tendency to report illusory stimuli in his left hemifield was modulated by masking of the right stimulus. When the mask was shown on the left (i.e., the right target was unmasked) the patient never reported the illusory stimulus (Figure 2). When the mask was bilateral, he reported 12 illusory stimuli (24%). Finally, when the mask was on the right he claimed seeing an illusory stimulus in 22 trials (44%). Thus, when a mask was used and the right stimulus was less visible, HG often perceived only the illusory stimulus. This finding supports the conclusion that the illusory item is triggered by early (possibly preconscious) visual processing, not by later recurrent processing (which is more likely to be perturbed by metacontrast masking). However, there is one complication to this conclusion: when the right item was shown unmasked the patient did not report the illusory left stimulus. This finding needs explanation, as it is contrary to all previous experiments (which did not use masked stimuli). We can exclude that the right colored target was more difficult to see than the black disk used in previous experiments, as HG detected it easily in unmasked conditions. It is more likely that the masking introduced more uncertainty and that HG was therefore more in doubt regarding the presence of a target. His access to phenomenological knowledge about the illusory stimulus (perceived as lighter and with later onset than the right item, see experiments 3 and 4), together with increased uncertainty about the presence of stimuli, may have led to a more conservative response criterion. So, why did the patient report an illusory stimulus only when the right item was masked? Masking of the right target hampered the elaboration of a high-level, conscious representation, but left the patient with a weak sensory trace of the stimulus. Under this condition, HG may have softened his decision criterion so that he started to report the illusory stimulus. In agreement with this interpretation is the striking difference between masking effects for stimuli in the right hemifield (11% detected when masked) versus the left hemifield (62% detected). This is what would be expected if HG softened his decision criterion, and possibly countered uncertainty through increased effort.

## 4. General Discussion

Our patient exhibited a peculiar visual illusion that has significant differences to palinopsia and polyopsia, as well as previous cases of visual allesthesia. Palinopsia is a visual illusion that often persists for seconds, minutes or up to days, and increases with increasing stimulus durations [12]. Patients often note the appearance of palinoptic images once the original stimulus has vanished and describe that they reach maximal intensity after several seconds [3,4,7]. Similarly, in most patients with polyopsia the illusory stimulus remains visible after disappearance of the original [13,14], and similarly descriptions of visual allesthesia indicate that the illusions persist over time [18,19]. Nevertheless, the symptoms of our patient come closest to the description of visual allesthesia, even though the illusory stimulus was only observed under specific experimental conditions and did not appear in free vision.

The illusory stimulus always appeared approximately at the homologous position in the hemifield contralateral to HG’s brain lesion, within a preserved part of his visual field. Its presence cannot be explained by a systematic strategy of the patient to report two stimuli whenever he saw one item in his right hemifield. Such a strategy would predict an increase of illusory stimuli for longer stimulus presentations, whereas HG showed the highest frequency of illusory reports at very short presentation time. Nevertheless, he had limited phenomenological knowledge of the illusory stimulus and made attempts to infer its visual characteristics such as shape. He perceived the illusion as fainter and as lagging behind the original stimulus. Interestingly, however, when shown one of two letters in his right hemifield, he believed that the illusory stimulus was the complementary letter. This finding is evidence for a strong top-down bias that leads HG to infer the shape of the illusory stimulus based on characteristics of the right-sided original. We believe that the cause for this bias must be sought in HG’s weak representation of the illusory stimulus. Since he perceived the left item as being lighter and delayed (Experiments 3 and 4), he had a strong feeling of ‘difference’ between the letter on the left and the letter on the right. Based on this feeling he therefore decided that the former must be different from the latter.

Several interpretations have been proposed to explain illusory phenomena in brain-injury patients. Critchley [1] noted an association with other visual impairments, in particular spatial confusion and deficits of localization. Similarly, Bender et al. [6] emphasized the ‘disorganized field of vision’ (p. 334) in their patients. Indeed, the clinical descriptions of many patients suggest the presence of visual localization deficits, difficulties in detecting and fixating objects, and occasionally optic ataxia, which are all clinical features reminiscent of Bálint syndrome [32,33,34]. When asked to point to different items on a sheet or a table these patients may point at positions that are not occupied by a stimulus; however, this symptom is most likely due to a mislocalization error, rather than the impression of an illusory stimulus. Our patient had some residual visual-spatial deficits, but these do not explain his illusory perceptions. In fact, based on his history of spatial neglect, we had expected him to exhibit visual extinction and were surprised to find that he was excellent at detecting unilateral left items even at very short presentation times. HG also never mislocalized visual stimuli, and his pointing or reaching performance was flawless.

Gersztenkorn and Lee [4] distinguished between hallucinatory and illusory palinopsia, and likened the former to a disturbance of visual memory and the latter to failures of visual perception. Though we have already pointed out the difference between our patient’s illusory perception and palinopsia, this proposal deserves consideration as possible explanation.

According to these authors perseveration of an original stimulus that has disappeared might be due to focal cortical hyperexcitability resulting in a release of visual memories, while illusions might reflect more diffuse alterations of cortical excitability and altered adaptation and feedback in visual pathways. Interestingly, the idea that illusions are signs of disinhibition of the visual cortex had already been formulated by Kinsbourne and Warrington [12], who surmised that, in palinopsia, parts of the visual cortex have been ‘released from some inhibitory controlling influence’ (p. 475). Newer neurophysiological studies allow identifying the mechanisms underlying the origins of cortical excitability with some precision. Of particular interest for our patient are the findings of neuroimaging studies examining cortical activation to unilateral visual stimulation [35,36]. A consistent finding of these studies is that retinotopic areas of occipital cortex that do not correspond to the position of the stimulus are deactivated, while areas responding directly to the stimulus are activated. Deactivation is particularly strong in the medial occipital cortex (corresponding to V1–V3), while it is less pronounced in the lateral occipital cortex [37,38]. In addition, the hemisphere ipsilateral to visual stimulation shows greater deactivation than the contralateral hemisphere [39]. Of particular significance is the finding that stimulus information can be decoded from deactivated regions, indicating that they retain a meaningful representation of the stimulus that has been presented to the contralateral hemisphere [40]. These findings suggest that a stimulus shown in one hemifield automatically activates visual cortices of both hemispheres. Homotopic coactivations and a high degree of correlated activity between homologous areas at rest appear to be a general feature of the brain, likely reflecting transcallosal facilitation [37,41]. However, in primary sensory areas such as V1, coactivation must be inhibited to prevent illusory perceptions. Evidence from combined transcranial magnetic stimulation (TMS) and fMRI studies indicates that the necessary inhibition of occipital excitability is provided through feedback projections from parietal or frontal areas to visual cortex. One such finding is that stimulation of the frontal eye fields (FEF) with TMS leads to increased activity in the peripheral visual field and decreased activity in more central regions throughout visual areas V1–V4 [42]. Even more significant, TMS of the intraparietal cortex modulates activity in early visual cortex only if no stimulus is present [43]. Together, these findings suggest that homotopic activations of contralateral visual cortex may be modulated through connections from the parietal cortex [44].

This model of transcallosal activation and modulation of visual cortex through feedback projections provides a plausible physiological explanation for illusory stimuli reported by HG. According to this model HG experiences automatic activation of visual cortex ipsilateral to the original stimulus as weak visual impressions at homotopic regions of the contralateral hemifield. Owing to his damage to the right posterior parietal cortex, these visual impressions are released from modulating inhibition and are therefore perceived as illusory stimuli. As supported by the masking experiment, the illusory stimulus is triggered by early, feedforward visual activity and therefore quickly vanishes due to lack of recurrent visual processing.

In conclusion, we propose that illusory stimuli may indeed be due to excitation of the visual cortex, as originally suggested by Kinsbourne and Warrington [12], as well as other authors. We add to this explanation the notion of top-down modulating feedback from the parietal to the occipital cortex, which may selectively be decreased or disrupted by parietal damage. Our study thus provides a plausible mechanistic explanation of a phenomenon that, at the first glance, appears bizarre and paradoxical.

## Figures and Tables

**Table 1:** HG’s scores in visual and visual-spatial tests.

Test Battery		Score	Cutoff/Percentile
Bells test ^1^	Left omissions	0	<cutoff
	Right omissions	0	<cutoff
Inversed T ^2^	Left omissions	0	<cutoff
	Right omissions	0	<cutoff
Letter cancellation ^3^	Left omissions	0	<cutoff
	Right omissions	0	<cutoff
Reading ^4^	Left transformations	0	<cutoff
	Right transformations	0	<cutoff
Simple reaction time ^5^	Without warning tone, ms	242	34
	With warning tone, ms	235	34
Choice reaction time ^5^	Reaction time, ms	434	46
Sustained attention ^5^	Reaction time, ms	770	12
	Omissions	12	42
Spatial cueing task ^5^	Left valid, ms	441	**7**
	Left invalid, ms	403	34
	Right valid, ms	267	90
	Right invalid, ms	290	93
Visual search ^5^	Reaction time, ms	4179	**5**
	Missed	7	42
Line bisection ^6^	Deviation (percent)	3.59	**4**
Rey figure ^7^	Copy, correct	24	**>1**
	Copy, shifted	0	59
	Copy, deformed	8	**1**

Note: ^1^ [20]; ^2^ [21]; ^3^ [22]; ^4^ [23]; ^5^ [24]; ^6^ [25]; ^7^ [26].
